# Reintegration programmes for people with severe mental illnesses released from correctional institutions: systematic review

**DOI:** 10.1192/bjo.2026.12034

**Published:** 2026-07-08

**Authors:** Alexander I. F. Simpson, M. Umer Waqar, Cory Gerritsen, Vito Adamo, Marco Kilada, Margaret Maheandiran, Reena Besa, Roland M. Jones

**Affiliations:** Forensic Psychiatry Division, Centre for Addiction and Mental Health and University of Toronto, Canada; Temerty Faculty of Medicine, University of Toronto, Canada; Department of Education, Mental Health Sciences Library, Centre for Addiction and Mental Health and University of Toronto, Canada

**Keywords:** Forensic psychiatry, correctional psychiatry, reintegration, recidivism, psychiatry and law

## Abstract

**Background:**

People with severe mental illness (SMI) face significant challenges when reintegrating from correctional facilities back to the community. These include increased recidivism risk, homelessness and mental health deterioration. Although specialised reintegration programmes were developed to address the needs of this vulnerable population, their effectiveness remains unclear owing to mixed findings reported in the literature.

**Aims:**

We performed a systematic review to synthesise and evaluate available evidence on reintegration programmes designed specifically for people with SMI.

**Method:**

Studies were included if they focused on adults with SMI transitioning from correctional facilities to community settings. The search was conducted through 10 January 2026. Two reviewers independently screened eligible studies and scored the appropriate risk of bias tools under supervision. A narrative analysis was adopted given the heterogeneity of methodologies and outcomes.

**Results:**

We identified 26 eligible studies, of which 11 were longitudinal, seven were randomised controlled trials, 3 had an AB design, 2 quasi-experimental, 2 retrospective and 1 brief report. The median number of participants involved in these studies was 207 (range: 22–3086). Studies employed various interventions, including peer mentoring, critical time intervention models, case management and therapeutic community. Results showed mixed effectiveness: some programmes demonstrated reduced recidivism and improved mental health service engagement, whereas others had minimal impact or even counterintuitive increases in re-imprisonment. Programmes generally showed better outcomes with a longer duration and comprehensive aftercare components.

**Conclusions:**

Reintegration programmes show promise, but current approaches demonstrate variable efficacy. Successful interventions appear to require comprehensive, sustained support addressing multiple domains including mental health treatment, housing, employment and social support.

The reintegration of people with severe mental illness (SMI) back into society following incarceration presents significant challenges for the people concerned, correctional systems, mental health service providers and society as a whole. Following their release, these individuals encounter many challenges including stigma, homelessness, unemployment and difficulty accessing adequate mental health services.^1,2^ Without appropriate assistance, they are at an elevated risk of mental health deterioration, hospital admission, recidivism and re-incarceration.^3^

Given the complexity of reintegration tasks (including a complex interplay of intrapersonal, interpersonal, institutional and community-level factors), specialised reintegration programmes have been developed that coordinate interventions across these domains to achieve successful outcomes.^4^ Such programmes can contain facilitated access to healthcare and addiction services, assistance with social supports such as housing, financial support and employment.^5,6^ However, the effectiveness of such interventions for people with SMI is unclear given the variability in interventions and mixed results reported in the literature.

A meta-analysis by Martin et al found that interventions targeting justice-involved individuals with mental disorders reduced criminal justice involvement, although the overall effect size was small with only modest improvements reported in mental health symptoms and functioning of participants.^7^ Hopkin et al found that reintegration programmes can improve contact with mental health services, but the impact on re-incarceration is complex, and it can paradoxically lead to increased re-incarceration.^8^

Persons with SMI transitioning into the community face significant physical health challenges such as increased risk of hepatitis C, cirrhosis and diabetes. These conditions, combined with other issues such as accidental overdose and increased risk of suicide, make this population particularly vulnerable to adverse outcomes and heightened risk of death within the first 2 weeks after release.^9–11^ Successful reintegration entails collaboration between several agencies to ensure continuous support during this critical transition juncture.^12^

This updated systematic review aims to provide a comprehensive synthesis of reintegration studies for individuals with SMI transitioning into the community from correctional settings. Unlike previous reviews that were limited in scope^7^ or did not include the most recently published studies,^8^ this review broadens the search to include multiple academic databases and grey literature. This review attempts to identify effective practices, highlight existing knowledge gaps and inform future directions for research, policy and service development in correctional mental healthcare.

## Method

This systematic review adheres to the Preferred Reporting Items for Systematic Review and Meta-Analyses (PRISMA) Statement.^13^ A protocol was developed and registered with PROSPERO (identifier: CRD42022299983) on 4 February 2022.^14^

### Information sources and search strategy

A search strategy was developed in conjunction with a medical librarian (R.B.). We searched Ovid Medline, Ovid PsycINFO, Ovid EMBASE, Ebsco CINAHL, ProQuest’s Applied Social Sciences Index and Abstracts (ASSIA), Ebsco Criminal Justice Abstracts and Cochrane CENTRAL, from inception until the end of November 2025. No search filters or limits were applied to the search strategy. The complete search syntax for all databases searched is provided in Supplementary Appendix A. Grey literature – including dissertations and conference abstracts – was identified via database searches and targeted screening of key Canadian and American government agencies (federal, state and provincial) to capture emerging research. This search focused on organisations specialising in justice, corrections, public safety, social service, mental health and re-entry services. All identified materials were screened using the same inclusion and exclusion criteria applied to peer-reviewed literature.

### Eligibility criteria

Inclusion and exclusion criteria for this review were structured using the Population, Intervention, Comparison, Outcome and Study (PICOS) Framework. The population of interest included adults (≥18 years) who were in a correctional environment (prisons, jails or detention centres), specifically those with an SMI who were released into the community under any conditions following any period or form of incarceration. Excluded populations were youth and adolescents, individuals found not criminally responsible or unfit to stand trial, those in psychiatric hospitals (including hospitals embedded within correctional systems) and populations without SMI symptoms, e.g. those with intellectual disabilities, sex offenders or those with only personality or substance misuse disorders.

Interventions considered were psychological, pharmacological, social work or other mental health interventions that commenced within correctional settings and aimed to improve mental health outcomes after release, such as reducing symptoms, improving functioning or lowering rates of re-incarceration. Interventions that commenced only after release were excluded. Those targeting concurrent disorders (e.g. substance use and mental health) were included if mental health outcomes were primary. Diversion programmes, like mental health courts, were also included. Comparisons included any design with or without a control group, such as pre–post studies, case-matched controls, or treatment-as-usual (TAU). Outcomes of interest were broad and included symptoms of SMI, psychological distress, well-being, healthcare utilisation, treatment adherence and motivation, social functioning, and rates of re-offence or re-incarceration, with any measures of effect accepted.

Eligible study designs included randomised controlled trials (RCTs), quasi-experimental, cohort, case–control, cross-sectional, case and brief reports with data, qualitative studies with data and opinion pieces with data, provided they were available in English or translated. Systematic reviews, meta-analyses, literature reviews and scoping reviews were excluded. Commentaries, editorials and opinion pieces without data were also excluded.

### Study selection, data collection process and data synthesis

We imported references into Covidence (a web-based collaboration software platform that streamlines the production of systematic and other literature reviews) and duplicates were automatically removed. Two independent reviewers (V.A. and M.K.) screened titles and abstracts before full-text screening. Any conflicts between the two reviewers that arose were communicated to a senior scientist (A.I.F.S., C.G. or R.M.J.), who resolved these and provided the final decision. A fifth of all included studies, those that made it to the full-text review stage, were also reviewed by the senior scientists to ensure reliability; the scientists and reviewers were in agreement.

**Fig. 1:**
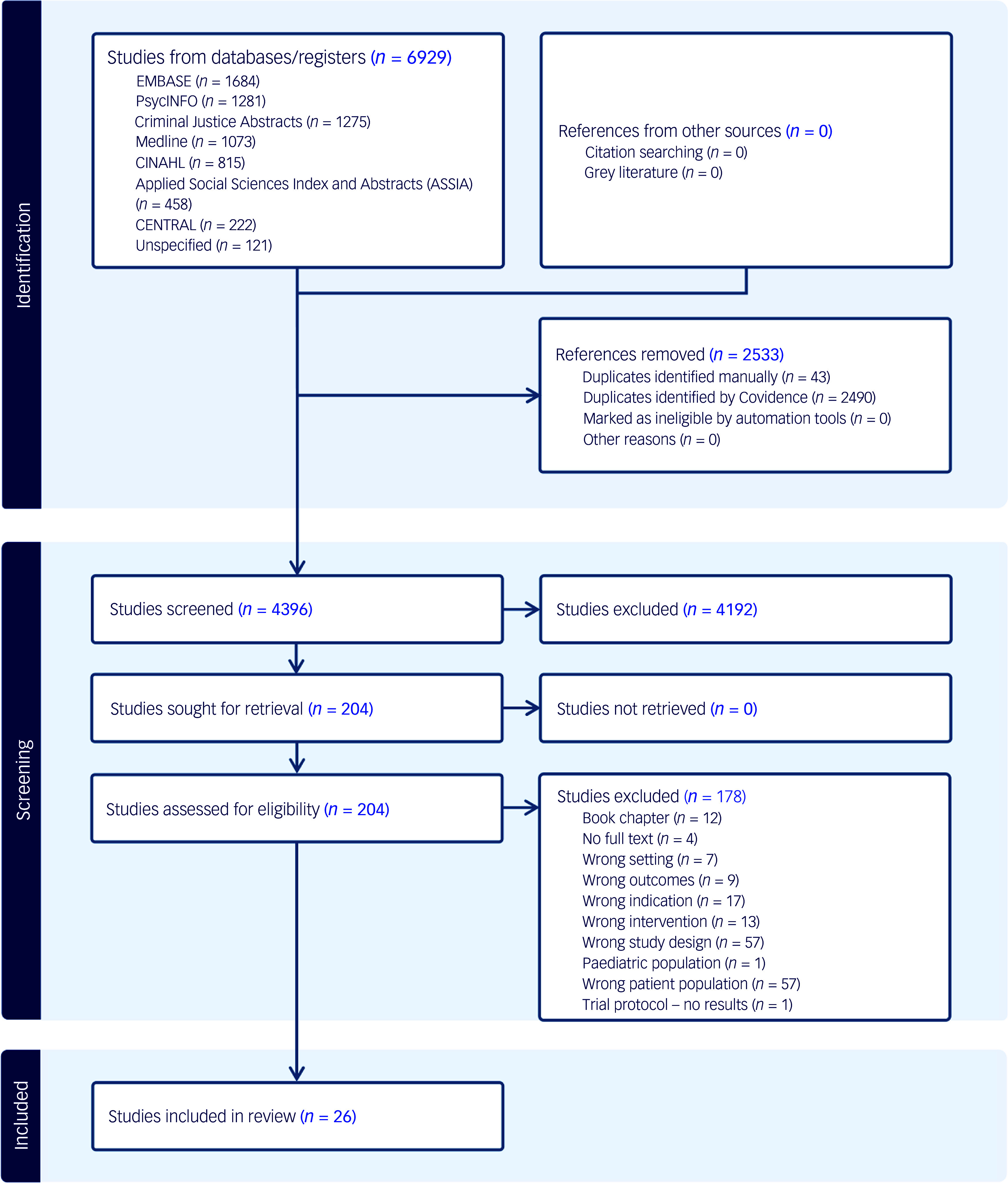
Preferred Reporting Items for Systematic Review and Meta-Analyses flow diagram.

Full-text screening was independently conducted by two reviewers (V.A. and M.K.). Any conflicts that arose during this phase were resolved by a senior scientist (A.I.F.S., C.G. or R.M.J). After the full-text screening phase concluded, data extraction using standardised data extraction forms first commenced on 8 February 2024, after the original search. Data extraction was conducted independently, in duplicate, by two reviewers (V.A. and M.K.). Any conflicts that arose during the extraction process were resolved through discussion between the pair, and if consensus could not be reached, a senior scientist (A.I.F.S., C.G. or R.M.J.) was consulted to provide a final decision. An updated search was conducted through 10 January 2026. Title and abstract screening as well as full-text screening following the updated search was conducted by three reviewers (M.U.W., A.W. (independent reviewer) or C.P. (independent reviewer)), with two votes being needed for study inclusion or exclusion. Any conflicts that arose were resolved after discussion with a senior scientist (A.I.F.S. or R.M.J.). No new studies met the eligibility criteria to proceed to the data extraction phase following the updated search. The screening process is summarised in the PRISMA flow diagram (Fig. 1).

### Study risk-of-bias assessment

The Risk of Bias in Non-randomised Studies of Interventions (ROBINS-I) was used to evaluate risk of bias in non-randomised intervention studies; this tool has seven domains: confounding, participant selection, intervention classification, deviations from intended interventions, missing outcome data, outcome measurement and selective reporting of results.^15^ The Cochrane Risk of Bias Tool (RoB 2) was used to evaluate risk of bias in RCTs, and includes five domains: randomisation, deviations from intended interventions, missing outcome data, measurement of the outcome and selection of the reported results.^16^ Risk of bias was assessed for each article by one junior reviewer (V.A., M.K. or M.U.W.) and independently by one senior scientist (A.I.F.S., C.G. or R.M.J.); any differences that arose were resolved through discussion between the pair. Risk of bias plots were generated using the *robvis* tool.^17^

## Results

### Study selection and characteristics

**Fig. 2:**
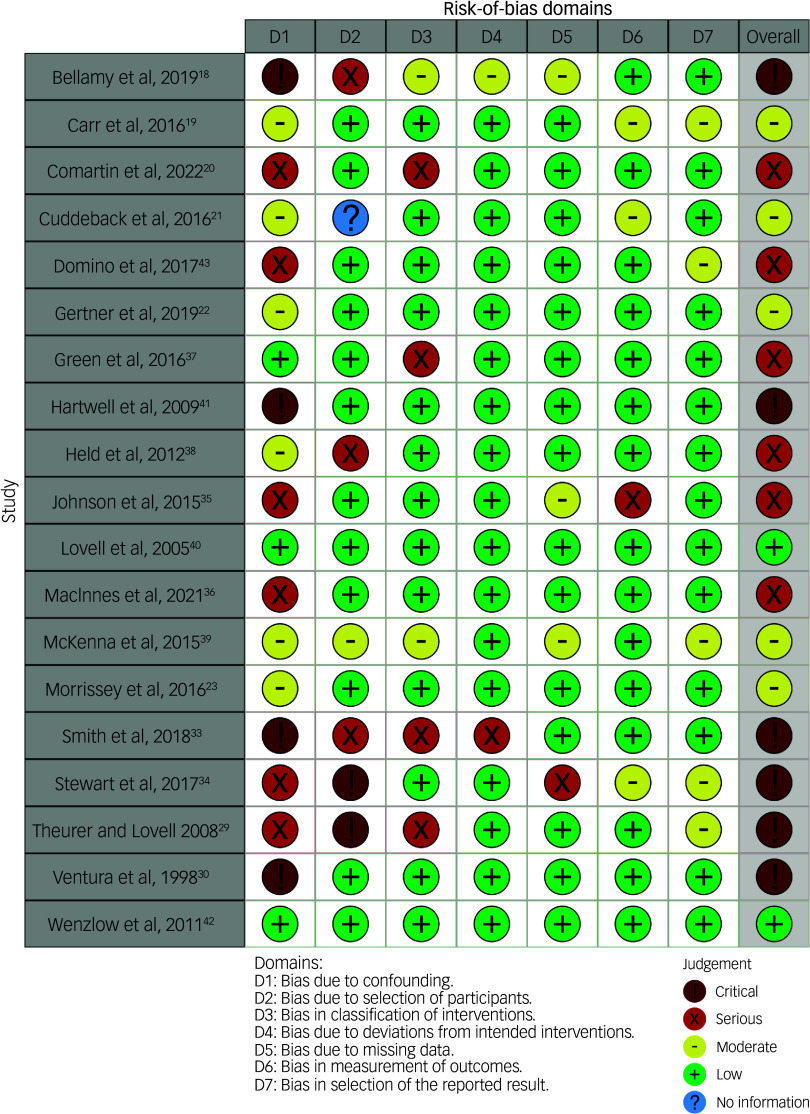
Risk-of-bias assessments for non-randomised controlled trials, using the Risk of Bias in Non-randomised Studies of Interventions tool.

**Fig. 3:**
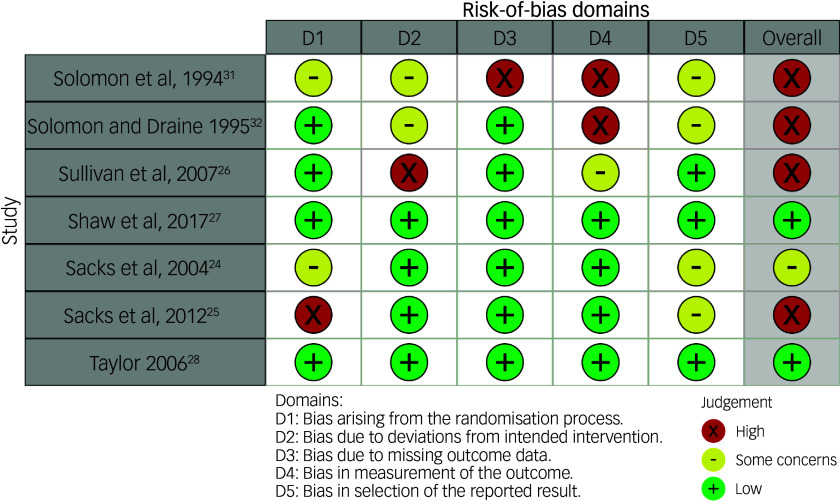
Risk of bias assessments for randomised controlled trials, using the Cochrane Risk of Bias Tool.

We identified 6929 studies, of which 2533 were duplicates, leaving 4396 studies. Following eligibility screening, 26 studies were deemed eligible for inclusion, from which we extracted data. Most of the included studies were from the USA (*n* = 21), followed by the UK (*n* = 2), and one each from Australia, Ireland and New Zealand. The median number of patients involved in these studies was 207 (range: 22–3086). Eleven studies were longitudinal, seven were RCTs, three had an AB design, two were quasi-experimental, two were retrospective and one was a brief report. Figures 2 and 3 report the risk of bias scores for each individual study. The results of the ROBINS-I tool assessments indicate varying levels of bias risk across non-RCT studies (Fig. 2); somewhat similar results were obtained using the RoB 2 tool for RCTs included in the review (Fig. 3).

### Study findings and outcomes

Tables 1–3 summarise the studies of community reintegration programmes for released prisoners with SMI. The interventions used encompassed peer support,^18^ day reporting centres,^19^ pre- and post-release mental health services,^20^ expedited Medicaid enrolment,^21–23^ modified therapeutic communities,^24–26^ critical time intervention method,^27^ intensive case management^28,29^ and community-based teams.^30–33^ Evaluations of different interventions highlighted variable degrees of success in terms of the outcomes measured, which were recidivism, enrolment in and engagement with mental health services, psychiatric symptoms and social functioning.

**Table 1:** Community support service interventions Table 1 long description.

Study	Study method	Sample characteristics and location	Description of intervention	Outcome	Risk of bias
Bellamy et al, 2019^18^	Retrospective	300 prisoners (mixed male and female); 79% had SMI; mean age: 32 years; USA	Peer mentoring for >2 years; some participants tracked for >1000 days. Measured re-incarceration rate	Lower re-incarceration (22%) compared with the general prison population (43%) within the first year of release	Critical
Carr et al, 2016^19^	Longitudinal/cohort study	613 probationers with mental illness (mixed); mean age: 34.64 years; 227 matched sample; USA	Day reporting centre: vocational, accommodation and psychiatric support	Lower reconviction rates (49%) compared with the comparison group (69%) and longer time to recidivism	Moderate
Shaw et al, 2017^27^	Randomised controlled trial	150 male prisoners (CTI: 72 and TAU: 78); mean age: 36.3 years; SMI criteria included; UK	CTI: intensive to meet health, social care and resettlement supports; data collection: 6 weeks, 6 months and 12 months after release	Engagement with services higher at 6 weeks for the intervention group (53%) compared with TAU (27%); sustained at 6 months; no differences at 12 months	Low
Smith et al, 2018^33^	Longitudinal/cohort study	43 sentenced male prisoners (comparison: 8); mean age: 36 years; SMI criteria included; Ireland	Pre-Release Planning programme: case management by social workers; focused on building trust, coordinating with agencies, offering advocacy and release planning	Accommodation and mental health support improved post-release; 46.5% re-incarcerated; no correlation between re-incarceration and improvements	Critical

SMI, severe mental illness; CTI, Critical time intervention; TAU, treatment as usual.

**Table 2:** Community mental health service interventions

Study	Study method	Sample characteristics and location	Description of intervention	Outcome	Risk of bias
Comartin et al, 2022^20^	Longitudinal/cohort study	1183 prisoners with mental illness (mixed); mean age: 37 years; USA	Pre- and post-release interventions focused on early release advocacy, in-jail treatment, discharge planning and post-discharge services.	Diversion and high-dose programme participants, especially completers, had higher treatment engagement rates, higher care continuity and treatment engagement; no impact on recidivism.	Serious
Green et al, 2016^37^	Longitudinal/cohort study	63 prisoners (mixed) with SMI (TR-short: 30, TR-long: 23; TCP only (control): 10); mean age: 30.3 years; Australia	TCP offered clinical mental health support; TRRanS provided community-based assistance (life skills, housing, finances, and service connections).	TRRanS reduced re-incarceration risk. TR-long participants had 40% lower risk of re-incarceration compared with TR-short participants; TCP-only participants had a 65% lower risk than TR-short.	Serious
Hartwell et al, 2009^41^	Brief report	799 prisoners (mixed) with SMI; post-regionalisation episodes: 432; pre-regionalisation episodes: 525; USA	Regionalisation of forensic transition teams, offering re-entry transition starting 3 months pre-release: identify service needs and commence benefit reinstatement process.	Post-regionalisation engagement increased for county corrections (59 *v*. 49%) and state prisons (44 *v*. 31%); re-incarceration rates rose for both (24 *v*. 10%; 23 *v*. 4%). Lost to follow-up rates were mixed.	Critical
Held et al, 2012^38^	AB design	207 homeless prisoners (mixed) with SMI; mean age (at first contact): 39.3 years; USA	Individual and group counselling, and telepsychiatry; case managers provided pre-release transition planning services.	2x2 analysis of variance demonstrated significant post-engagement decreases in recidivism: annual bookings (down 57.1%), charges (down 57.4%), felonies (down 71%) and misdemeanours (down 49.5%).	Serious
Johnson et al, 2015^35^	AB design	22 incarcerated women with SUD and MDD; mean age: 36 years; USA	Sober Network Interpersonal Therapy: pre-release in-prison group and individual sessions focusing on substance use, mental health, sober support networks and depression resolution.	Depressive symptoms and social support improved, reductions in drinking and drug-using days, with days of abstinence increasing during the study period.	Serious
Lovell et al, 2005^40^	Longitudinal/cohort study	387 (DMIO: 100, CTS: 287) prisoners (mixed) with SMI; mean age (DMIO): 37 years; USA	Washington State’s DMIO law provides comprehensive support services; comparison group: CTS.	DMIO programme reduced overall and felony recidivism, but not violent felony recidivism; DMIO participants received immediate and comprehensive mental health services and housing support.	Low
MacInnes et al, 2021^36^	Longitudinal/cohort study	61 (31 intervention, 31 comparison) male prisoners with mental illness, personality disorder and SUD; UK	RESET programme offered comprehensive release-day support, which was compared with standard care.	RESET group had lower homelessness, short-term reoffending and higher service engagement across mental health, primary care and benefits access; no differences in long-term reoffending.	Serious
McKenna et al, 2015^39^	AB design	278 (pre-intervention: 108); post-intervention: 170) prisoners (mixed) with SMI; New Zealand	PMOC: screening, triage and community engagement; pre-release planning began 3 months before, with system-wide screening and post-release mental health service engagement.	PMOC implementation increased engagement with community services across groups and follow-up contact with mental health and other services; non-significant decrease in reoffending.	Moderate
Sacks et al, 2004^24^	Randomised controlled trial	185 male prisoners (MTC programme: 92; mental health programme: 93) with SMI and SUD; mean age: 34.29 years; USA	MTC programme compared with a mental health programme, both offering pre-parole prison interventions followed by aftercare.	MTC participants, especially those completing aftercare, had lower re-incarceration and criminal activity (overall and substance-related); no difference in time until re-offence.	Some concerns
Sacks et al, 2012^25^	Randomised controlled trial	127 male prisoners (intervention: 71; comparison: 56) with mental illness and SUD; mean age: 38.2 years; USA	Experimental Re-entry Modified Therapeutic Community: cognitive behaviour-based residential programme for criminogenic thinking, substance misuse and mental illness.	Participants had lower rates of re-incarceration (19 *v*. 38%), criminal activity (39 *v*. 62%) and substance-related offences (37 *v*. 58%).	High
Solomon et al, 1994^31^	Randomised controlled trial	200 (ACT: 60, FICM: 60, CMHC: 80) prisoners (mixed) with SMI and personality disorder; mean age: 35.4 years; USA	ACT teams provided comprehensive support; FICMs worked independently; both compared with standard CMHCs.	32% returned to jail 6 months after release; ACT team clients had higher re-incarceration rates (60%) compared with FICM (35%) or CMHC (40%); lack of self-care training programmes significantly correlated with re-incarceration.	High
Solomon and Draine, 1995^32^	Randomised controlled trial	94 (ACT: 37, FICM: 35, CMHC: 22) male prisoners with SMI and personality disorder; mean age: 34.9 years; USA	ACT teams provided comprehensive support; FICMs worked independently; both compared with standard CMHCs.	46% of participants returned to jail within a year, with more ACT team clients (60%) re-incarcerated compared with FICM (40%) or CMHC (36%), albeit differences not statistically significant.	High
Stewart et al, 2017^34^	Longitudinal/cohort study	646 (65 CDP only, 249 CMHS only, 63 CDP + CMHS, 269 non-CMHI) male prisoners with mental illness and personality disorder; USA	CMHI offers four different services: CDP only, CMHS) only, a combination of both CDP and CMHS, and TAU (non-CMHI).	CMHS only group had lower re-incarceration rates (at months 3 and 6) and lower reoffending (months 24 and 48); no differences in violent recidivism between groups.	Critical
Sullivan et al, 2007^26^	Randomised controlled trial	185 (intervention: 92, comparison: 93) male prisoners with mental illness and SUD; mean age: 34.3 years; USA	MTC for mental disorders and SUD; participants randomly assigned to either MTC or a control group with standard treatment.	Both groups improved in mental health symptoms, with increases in medication use and treatment involvement. Substance use relapse predicted criminal activity and re-incarceration.	High
Taylor, 2006^28^	Randomised controlled trial	206 (PALS: 106, TAU: 100) prisoners (mixed) with SMI; mean age: 35.88 years; USA	PALS programme offered intensive case management and support; participants randomly assigned to the PALS group or TAU.	PALS group utilised more individual counselling until 180 days, but not afterward; no differences in recidivism rates between the two groups.	Low
Theurer and Lovell, 2008^29^	Retrospective matched control design	1426 (intervention: 64, control: 1362) prisoners (mixed) with mental illness; mean age at release: 35.9 years; USA	Intensive case management treatment programme, including pre-release planning, intensive post-release case management and residential support services.	Programme participants had lower felony recidivism rates compared to controls (23 *v*. 42%). McNemar test results show recidivism reduction particularly for felonies after the first year.	Critical
Ventura et al, 1998^30^	Longitudinal/cohort study	261 (intervention: 203, comparison: 58) prisoners (mixed) with mental illness; mean age: 30 years; USA	Case management services provided in prison and post-release, including diagnostic assessment, counselling, crisis intervention, psychiatric evaluation, medication and community re-entry planning.	72% rearrested within 3 years. Prison-based case management indirectly lowered re-arrest probability and lengthened time to re-arrest by increasing likelihood of community-based case management receipt.	Critical

TR-short, short periods (<62 days); TR-long, longer periods (>62 days); SMI, severe mental illness; TCP, Transitional Coordination Programme; TRRanS, Transition Reintegration Recovery and Support; SUD, substance use disorder; MDD, major depressive disorder; DMIO, Dangerous Mentally Ill Offender; CTS, Community Transition Study; RESET, RESETle programme; PMOC, Prison Model of Care; MTC, Modified Therapeutic Community; ACT, assertive community treatment; FICM, forensic individual case manager; CMHC, community mental health centre; CDP, clinical discharge planning; CMHS, community mental health service; CMHI, Community Mental Health Initiative; TAU, treatment as usual; PALS, Providing Assistance with Linkage to Services.

**Table 3:** Health insurance access-related interventions Table 3 long description.

Study	Study method	Sample characteristics and location	Description of intervention	Outcome	Risk of bias
Cuddeback et al, 2016^21^	Quasi-experimental study	998 (out of 1248 referred) prisoners and psychiatric hospital patients with SMI (mixed); mainly age 26–45 years; USA	Expedited Medicaid applications, and connections with supports. Out-patient service utilisation measured at 30, 60 and 90 days	Those approved (more likely White, female, schizophrenia or bipolar, or previous Medicaid) had higher Medicaid enrolment (89 *v*. 56%) and mental health service use (71 *v*. 39%) 30 days post-release	Moderate
Domino et al, 2017^43^	Longitudinal/cohort study	3086 prisoners (referred: 871; comparison: 2215) with SMI (mixed); mean age: 36.25 years; USA	Expedited Medicaid referrals; follow-up 90 days post-release; mental health treatment and criminal justice involvement measured, including arrests 12 months later	>50% of released individuals re-arrested within a year; referred group had more prison stays; treatment levels associated with increased re-incarceration probability	Moderate
Gertner et al, 2019^22^	Longitudinal/cohort study	3086 adults (mixed) with SMI released from prison (referred: 871; comparison: 2215); mean age: 36.5 years; USA	Expedited Medicaid referrals within 90 days of release; primary outcome: SUD treatment use within 3, 6 and 12 months	Referrals increased likelihood of treatment use at 3, 6 and 12 months and case management receipt at months 3 and 6, but not 12	Moderate
Morrissey et al, 2016^23^	Quasi-experimental study	3086 offenders (mixed) with SMI released from state prisons (intervention: 895; comparison: 2191); mean age: 36.25 years; USA	Intervention: pre-release referral for expedited Medicaid enrolment; primary outcomes measured at release, after 30 days and within 12 months	Referred group had higher Medicaid enrolment at release, 30 days and 12 months (*p* < 0.01); higher service use, but more prison days despite no arrest rate differences	Moderate
Wenzlow et al, 2011^42^	Longitudinal/cohort study	686 prisoners (mixed) with SMI (project facilities: 272 (treatment: 77; baseline: 195); comparison facilities: 414 (treatment: 130; baseline: 284)); USA	Oklahoma’s discharge planning: early identification, consent and assistance with federal disability and Medicaid applications	Release day Medicaid enrolment increased in treatment group (25% from 8% 2 years ago; 3–4% in comparison facilities); sustained at 30, 60 and 90 days	Low

SMI, severe mental illness; SUD, substance use disorder.

As detailed in Supplementary Appendix B, the operational definitions of both SMI and recidivism varied considerably across the studies reviewed. Diagnoses classified as SMI generally centred on schizophrenia and bipolar disorder, but frequently expanded to include others, depending on the study’s inclusion criteria. Recidivism was mainly defined as re-incarceration, although some studies included technical parole violations whereas others strictly measured new offences.

Studies can be broadly grouped into three categories based on the type of intervention employed: linkage to community support services, linkage or provision of community mental health services, and health insurance access interventions. We will discuss each in turn.

### Community support services

Studies in this category aimed to link individuals to community support services. These include a single RCT, two longitudinal cohort studies and one retrospective study (see Table 1). Two studies had a critical risk of bias,^18,33^ one had moderate risk^19^ and one RCT had a low risk.^27^ Four studies demonstrated generally positive impacts on service engagement and, in some cases, recidivism reduction. Bellamy et al found that peer mentoring commenced before release and continued in the community was associated with lower re-incarceration rates (22%) in the first year after release compared with the general prison population (43%).^18^ Carr et al reported that participants in a day reporting centre with integrated vocational, accommodation and psychiatric support had lower reconviction rates (49%) and lower longer time to recidivism than matched controls (69%); completion status within the day reporting centre programme also predicted time to any conviction.^19^ Shaw et al conducted an RCT of a critical time intervention model and observed improved and more consistent contact with community mental health services after release, although the reduction in reoffending was not sustained at later follow-up.^27^ Smith et al, in a study on the Pre-Release Planning programme, found that a substantial number of participants (46.5%) were re-incarcerated during the study period, with no significant correlation between re-incarceration and improvements in mental health support or accommodation.^33^ These studies highlight that although case management and community support interventions can enhance continuity of care and initial service engagement, their effects on recidivism and long-term criminal justice outcomes remain mixed.

### Community mental health services

Studies on linkages directly to community mental health service teams showed some positive results (see Table 2). The highest quality of evidence came from two RCTs, which investigated modified therapeutic communities and reported positive findings in terms of recidivism reduction. The modified therapeutic community group had lower re-incarceration rates (9%) than the comparison group (33%) in one study; in the other study, the experimental re-entry modified therapeutic community group had lower re-incarceration rates (19%) than the control parole supervision case management group (38%).^24,25^ However, in terms of risk of bias, these studies had some concerns^24^ or a high risk.^25^ On the other hand, two RCTs showed no significant difference in rate of re-incarceration among people under assertive community treatment, forensic individual case managers or the community mental health centre; both these RCTs had a critical risk of bias.^31,32^ Another two RCTs, one of a modified therapeutic community (high risk of bias) and another of a service-linkage programme (low risk of bias), reported mixed findings with periodic crisis stabilisation, increased service utilisation and some symptom improvement, but no significant differences were found in measures of symptom change and recidivism between groups.^26,28^

Non-RCT study methods in this category included longitudinal/cohort studies, AB design, retrospective matched control design and brief report. Four studies had a critical risk of bias,^12,29,30,34^ five had a serious risk,^20,35–38^ one had a moderate risk^39^ and one had a low risk.^40^ The Sober Network cellphone-based intervention results suggested better social support and a reduction in depressive symptoms and substance use on community follow-up of women with substance use disorders and major depression.^35^ A study revealed that participants in an intensive case management treatment programme had a recidivism rate of 23%, which was just over half of the 42% rate observed in matched controls.^29^ In another study, men receiving only community mental health services had lower risk of recidivism and returning to custody.^34^ Two of these had a critical risk of bias^29,34^ and one had a serious risk.^35^ Another study, with a serious risk of bias, found no significant association between specific programme characteristics and recidivism.^20^ Additionally, increased return to the criminal justice system was observed after regionalisation of forensic transition teams in one study with a critical risk of bias.^41^

With regard to service engagement and continuity of care, diversion participants, high-dose programme participant, and programme completers were more likely to engage in treatment both within 14 days and a year post-release, compared with non-diversion participants, low-/medium-dose programme participants and non-completers;^20^ this indicates that continuity of care was significantly influenced by programme dosage and completion. The Dangerous Mentally Ill Offender programme in the USA was noted to succeed in providing more comprehensive and consistent mental health services, and reducing overall and felony recidivism, but not violent felony recidivism; this study had a low risk of bias.^40^ The development of a Prison Model of Care was studied using an AB design, which was focused on enhanced case detection, assessment and engagement of community mental health services before release in New Zealand. They found that men on remand achieved higher levels of engagement with general mental health services and observed a non-significant decrease in reoffending.^39^ The risk of bias in this study was moderate.

### Health insurance access-related interventions

Five studies fell into this category, which were either longitudinal cohort or quasi-experimental studies (see Table 3). Expedited Medicaid referrals were the most common intervention in this group. They are specific to jurisdictions with health insurance-based health services that require insurance enrolment to access healthcare follow-up and medication in the community. Such enrolment is frequently interrupted by incarceration. Four studies demonstrated that expedited Medicaid referrals were associated with higher enrolment rates and service utilisation post-release.^21–23,42^ Referral to expedited Medicaid enrolment predicted a higher probability of accessing any substance use disorder treatment.^22^ Also, Medicaid enrolment rates were significantly higher for approved individuals after 1 and 3 months following release.^21^ However, two studies reported mixed findings. One showed that this intervention, despite increased enrolment and service utilisation, did not reduce criminal recidivism.^23^ Another found that the group referred for expedited Medicaid had significantly more episodes of incarceration in the year following release.^43^ Four studies had a moderate risk of bias^21–23,43^ and one had a low risk of bias.^42^

## Discussion

We identified 26 studies that evaluated reintegration programmes for individuals with SMI transitioning from correctional institutions to the community. These varied widely in the type of interventions (e.g. peer support, transitional coordination, expedited Medicaid enrolment, integrated psychosocial interventions) and in outcomes measured (e.g. recidivism, treatment engagement, mental health symptoms, social functioning). Some programmes demonstrated efficacy in reducing recidivism and improving service engagement, the overall evidence base reflects a complex and rather mixed picture. Programmes addressing both mental health and social needs, such as those providing coordinated clinical care, housing and vocational support, were generally more effective in reducing recidivism and improving community engagement. For instance, peer mentoring and transitional coordination programmes consistently demonstrated lower rates of re-incarceration and higher treatment engagement rates compared with standard care;^18–20,37,38^ however, three of these studies had a serious risk of bias,^20,37,38^ one had a moderate risk^19^ and one had a serious risk.^18^ In contrast, programmes focusing solely on Medicaid enrolment or benefit reinstatement had mixed or even counterintuitive findings;^22,23,43^ some studies reported increased re-incarceration when Medicaid enrolment was not paired with clinical engagement or case management,^23,43^ suggesting that multicomponent interventions are more effective than single-focus programmes. However, there may be jurisdiction specific reasons for this, and the goal of achieving access to health benefits is of itself vital in the tasks of reintegration.

The findings for case management and clinical engagement align with prior research,^7^ demonstrating that interventions tackling both mental health-related factors and social determinants yield better outcomes than clinical or social interventions administered alone. The importance of addressing social determinants of health such as housing, financial support and employment alongside mental health treatment is well established in the literature.^44–46^ Peer support models, in particular, have gained recognition for their role in fostering engagement and stigma reduction.^47^ Integrated clinical and social support programmes are most consistent with existing evidence advocating for holistic approaches that address both mental health and social determinants. Although these programmes show promise, the evidence base is still limited, with small sample sizes and moderate-to-serious risk of bias in many studies.

Programmes focusing on expedited Medicaid enrolment or benefit reinstatement generally improved access to out-patient and substance use disorder treatment,^21,22^ but outcomes are mixed, especially with respect to re-incarceration.^8^ This may be because the programme was targeted too narrowly on health access alone, and not on a broad range of reintegration support. Instability may have been detected by health and probation services resulting in re-incarceration for breaches of release provision that might not otherwise have been detected.^48^ Thus, it is an attempt to provide more, but incomplete or siloed services may increase the risk of re-incarceration. Similar findings have been noted in studies of Forensic Assertive Community Treatment models for persons with SMI with a history of criminal justice involvement.^49,50^ Previous research indicates that, although insurance is necessary for access to care, coverage alone does not guarantee improved outcomes without coordinated clinical and social supports.^10,51^ Many programmes lacked randomised designs, thereby limiting causal inference.

Reintegration of prisoners with SMI into the community after release presents significant challenges that require comprehensive and coordinated approaches. This complex process involves addressing multiple interconnected factors, such as finances, employment, accommodation and mental healthcare continuity, to ensure successful outcomes for both the individuals and society at large.^2,52^ Studies are, therefore, understandably diverse and typically focus on a handful of issues; certain solutions also tend to be jurisdiction-specific (e.g. Medicaid in the USA). The availability and quality of mental health services and reintegration programmes varies greatly across jurisdictions, making it challenging to generalise findings. Conducting research into the reintegration of prisoners with SMI into the community presents several practical difficulties, which may include difficulties obtaining data because of privacy laws and bureaucratic obstacles, securing adequate funding for long-term studies, the logistical complexity of ensuring effective research collaboration between multi-disciplinary team staff and inconsistent definitions for ‘successful reintegration’ in different studies that make comparisons difficult.^52,53^

Traditional probation models usually focus on compliance instead of tackling underlying criminogenic needs or psychiatric symptomatology. This unidimensional approach can lead to higher recidivism rates compared with specialty models that integrate mental health treatment and supervision.^54^ Mental health service and probation linkages might initially appear to increase recidivism possibly as a result of increased detection of technical violations;^55^ however, determining true effectiveness of these services in the community often takes up to a year to achieve,^56^ complicating short-term evaluations. Although many included studies followed participants for a year or more, others focused on a much shorter duration, such as 3 months,^21^ 6 months^31,39^ and 9 months.^36^

Sources in this review offer diverse study designs across various jurisdictions, providing insights from different mental health and criminal justice systems. Nevertheless, heterogeneity of interventions and outcomes is also a weakness of this review, making direct comparisons and synthesis of findings even more challenging. Most studies (19 out of 26) were non-RCTs, consequently increasing the risk of selection bias and confounding. Long-term programme impacts are obscured by the fact that more than half of the included studies (17 out of 26) tracked outcomes for a year or less. All studies were conducted exclusively in high-income countries, limiting their applicability to low- and middle-income countries. Furthermore, as detailed in Supplementary Appendix B, because of considerable heterogeneity in how SMI and recidivism were defined across the literature, direct comparability of outcomes becomes limited; the thresholds for reintegration failure measurement differed significantly – such as whether technical violations were included or a new criminal charge alone constituted recidivism. Another limitation of this review is that inter-rater agreement statistics were not calculated and a Grading of Recommendations Assessment, Development and Evaluation (GRADE) assessment was not conducted, which could potentially affect the consistency of study inclusion decisions and evidence quality evaluation. In the present review, the included studies were highly heterogeneous in design, populations, interventions and outcome measures, and quantitative synthesis was not the primary objective; as such, applying GRADE would not have provided additional meaningful insight beyond the detailed risk-of-bias assessments performed already.

Successful reintegration of incarcerated individuals with SMI following release requires a multifaceted strategy that addresses both immediate and continued needs. The summarised studies reveal mixed outcomes, which highlights the complexity of addressing service engagement, social integration and recidivism among released prisoners with SMI. Although programme models and outcomes vary, interventions that offer sustained, coordinated support across clinical, social and structural domains, particularly those that integrate mental health care with housing, employment and peer support, tend to show the most promise in reducing recidivism and enhancing service engagement. In contrast, programmes narrowly focused on administrative tasks, such as expedited benefit enrolment without accompanying case management or therapeutic support, demonstrate limited or even counterintuitive impacts, including higher rates of re-incarceration likely linked to increased monitoring and detection of technical violations.

## Supporting information

10.1192/bjo.2026.12034.sm001 Simpson et al. supplementary material 1 Simpson et al. supplementary material

10.1192/bjo.2026.12034.sm002 Simpson et al. supplementary material 2 Simpson et al. supplementary material

## Data Availability

The authors confirm that the data supporting the findings of this study are available within the article.

## Supplementary material

The supplementary material is available online at https://doi.org/10.1192/bjo.2026.12034

## Table 1 Long description

A table summarizing studies on community reintegration programs for released prisoners with SMI. The table has 4 rows and 5 columns. Column headers are Study, Study method, Sample characteristics and location, Description of intervention, Outcome, and Risk of bias. Row 1: Study: Bellamy et al, 2019; Study method: Retrospective; Sample characteristics and location: 300 prisoners (mixed male and female); 79 percent had SMI; mean age: 32 years; USA; Description of intervention: Peer mentoring for greater than 2 years; some participants tracked for greater than 1000 days. Measured re-incarceration rate; Outcome: Lower re-incarceration (22 percent) compared with the general prison population (43 percent) within the first year of release; Risk of bias: Critical. Row 2: Study: Carr et al, 2016; Study method: Longitudinal/cohort study; Sample characteristics and location: 613 probationers with mental illness (mixed); mean age: 34.64 years; 227 matched sample; USA; Description of intervention: Day reporting centre; vocational, accommodation and psychiatric support; Outcome: Lower reconviction rates (49 percent) compared with the comparison group (69 percent) and longer time to recidivism; Risk of bias: Moderate. Row 3: Study: Shaw et al, 2017; Study method: Randomised controlled trial; Sample characteristics and location: 150 male prisoners (CTI: 72 and TAU: 78); mean age: 36.3 years; SMI criteria included; UK; Description of intervention: CTI: intensive to meet health, social care and resettlement supports; data collection: 6 weeks, 6 months and 12 months after release; Outcome: Engagement with services higher at 6 weeks for the intervention group (53 percent) compared with TAU (27 percent); sustained at 6 months; no differences at 12 months. Accommodation and mental health support improved post-release; 46.5 percent re-incarcerated, no correlation between re-incarceration and improvements; Risk of bias: Low. Row 4: Study: Smith et al, 2018; Study method: Longitudinal/cohort study; Sample characteristics and location: 43 sentenced male prisoners (intervention: 8; mean age: 36 years; SMI criteria included; Ireland; Description of intervention: Pre-Release Planning programme: case management by social workers; focused on building trust, coordinating with agencies, offering advocacy and release planning; Outcome: Not specified; Risk of bias: Critical.

Navigate back to Table 1.

## Table 3 Long description

A table summarizing studies on community reintegration programs for released prisoners with SMI. The table has six columns: Study, Study method, Sample characteristics and location, Description of intervention, Outcome, and Risk of bias. It contains multiple rows with specific details for each study. Row 1: Study: Cuddeback et al, 2016; Study method: Quasi-experimental study; Sample characteristics and location: 998 (out of 1248 referred) prisoners and psychiatric hospital patients with SMI (mixed); mainly age 26-45 years; USA; Description of intervention: Expected Medicaid applications, and connections with supports. Out-patient service utilisation measured at 30, 60 and 90 days; Outcome: Those approved (more likely White, female, schizophrenia or bipolar, or previous Medicaid) had higher Medicaid enrolment (89 vs. 56%) and mental health service use (71 vs. 39%) 30 days post-release; Risk of bias: Moderate. Row 2: Study: Domino et al, 2017; Study method: Longitudinal/cohort study; Sample characteristics and location: 3086 prisoners (referred: 871; comparison: 2215) with SMI (mixed); mean age: 36.25 years; USA; Description of intervention: Expedited Medicaid referrals; follow-up 90 days post-release; mental health treatment and criminal justice involvement measured, including arrest 12 months later; Outcome: >50% of released individuals re-arrested within a year; referred group had more prison stays; treatment levels associated with increased re-incarceration probability; Risk of bias: Moderate. Row 3: Study: Gernter et al, 2019; Study method: Longitudinal/cohort study; Sample characteristics and location: 3086 adults (mixed) with SMI released from prison (referred: 871; comparison: 2215); mean age: 36.5 years; USA; Description of intervention: Expedited Medicaid referrals within 90 days of release; primary outcome: SUD treatment use within 3, 6 and 12 months; Outcome: Referrals increased likelihood of treatment use at 3, 6 and 12 months and case management receipt at months 3 and 6, but not 12; Risk of bias: Moderate. Row 4: Study: Morrissey et al, 2016; Study method: Quasi-experimental study; Sample characteristics and location: 3086 offenders (mixed) with SMI released from state prisons (intervention: 895; comparison: 2191); mean age: 36.25 years; USA; Description of intervention: pre-release referral for expedited Medicaid enrolment; primary outcomes measured at release, after 30 days and within 12 months; Outcome: referred group had higher Medicaid enrolment at release, 30 days and 12 months (p < 0.01); higher service use, but more prison days despite no arrest rate differences; Risk of bias: Moderate. Row 5: Study: Wenzlow et al, 2011; Study method: Longitudinal/cohort study; Sample characteristics and location: 686 prisoners with SMI (project facilities: 272 (treatment: 193; baseline: 79); comparison facilities: 414 (treatment: 130; baseline: 284)); USA; Description of intervention: Oklahoma's discharge planning; early identification, consent and assistance with federal disability and Medicaid applications; Outcome: Release day Medicaid enrolment increased in treatment group (25% from 8% 2 years ago); 3-4% in comparison facilities; sustained at 30, 60 and 90 days; Risk of bias: Low.

Navigate back to Table 3.
