# Supplementary material for: Reintegration programmes for people with severe mental illnesses released from correctional institutions: systematic review
Source: BJPsych Open. 2026 Jul 8;12(4):e181. doi: 10.1192/bjo.2026.12034 (PMC13359055; doi:10.1192/bjo.2026.12034)
Supplement: Simpson et al. supplementary material 1 — Simpson et al. supplementary material [file S2056472426120341sup001.docx]

**Appendix A**

**Ovid MEDLINE: Epub Ahead of Print, In-Process & Other Non-Indexed Citations, Ovid MEDLINE® Daily and Ovid MEDLINE® <1946-Present>**

1 Mental Health/

2 exp Mental Disorders/

3 Mentally Ill Persons/

4 exp Psychiatry/

5 exp Forensic Psychiatry/

6 forensic*.mp.

7 ((mental* or psychiatr* or psychological*) adj3 (condition* or diagnos?s* or disorder* or health* or hygiene or ill* or safe* or wellbeing or well-being or wellness)).mp.

8 ((mental* or psychiatr* or psychological*) adj3 (complex or comorbid* or highly distress* or high needs* or impair* or intense or persisten* or re-admit* or readmit* or recurrence or recurrent or reoccur* or re-occur* or serious* or sever* or treatment resistant* or treatment-resistant)).mp.

9 exp "Schizophrenia Spectrum and Other Psychotic Disorders"/

10 schizo*.mp.

11 dementia praecox.mp.

12 Depressive Disorder, Major/

13 (major adj depressi* adj disorder*).mp.

14 (involutional adj (depressi* or melancholia or paraphrenia* or psychos?s)).mp.

15 exp Bipolar Disorder/

16 bipolar*.mp.

17 ((manic adj (disorder* or depress*)) or mania).mp.

18 psychos?s*.mp.

19 criminals/

20 criminal*.mp.

21 exp Prisoners/

22 Prisons/

23 prison*.mp.

24 penitentiar*.mp.

25 gaol*.mp.

26 jail*.mp.

27 (convict or convicts or convicted).mp.

28 offender*.mp.

29 remand*.mp.

30 sentence*.mp.

31 incarcerat*.mp.

32 (inmate or inmates or imprison*).mp.

33 (detain* not (immigrants or migrant* or refugee*)).mp.

34 ((detention adj3 (centre or center or facility or facilities or institution*)) not (immigrants or migrant* or refugee*)).mp.

35 forensic*.mp.

36 (pre-trial or pretrial or "pre trial").mp.

37 ("under trial" or under-trial or undertrial).mp.

38 (correction* adj3 (centre or center or facility or facilities or institution*)).mp. not (author correction or "correction to" or "Corrections To").ti.

39 (acquit or acquitted or acquittal or bail or "leaving jail" or (discharg* adj3 (correction* or custody or detention or incarcerat* or jail or prison*)) or parole* or probation or ((re-enter* or re-entre* or reenter* or reentre*) adj5 (community or communities or public or society)) or ((release or released) adj3 (correction* or custody or detention or incarcerat* or jail or prison*)) or ((reintegrat* or re-integrat* or re-settle* or resettle*) adj3 (community or communities or public or society)) or (transition* adj5 (correction* or custody or detention or incarcerat* or jail or prison* or community or communities or public or society))).mp.

40 Case Management/

41 Community Mental Health Centers/

42 Community Mental Health Services/

43 Mental Health Services/

44 Peer Group/

45 Psychiatric Rehabilitation/

46 Rehabilitation/

47 exp Social Support/

48 Social Work, Psychiatric/

49 Therapeutics/

50 ((alternative* or mental* or psychosocial* or psycho-social* or psychoeducation* or psycho-education* or resilient or resilience* or self efficacy or social or strength* based or wellbeing or well being or vocational or bridge or bridging or dual diagnos?s* or reentry) adj3 (care or counsel* or intervention* or maintenance or model or models or plan* or practice* or program* or service* or support* or system therapy or therapies or training or treatment or workshop*)).mp.

51 assertive community treatment.mp.

52 ((community or social or public) adj3 integrat*).mp.

53 (Aboriginal* or "First Nation" or "First Nations" or Indian or Indigenous or Inuit or Metis or Native or Natives or (Afro* or Afric* or Black or Blacks or ethnocultural or racialized) or (cultural* adj3 (appropriate* or approach or relevant* or specific*)) or ((overrepresent* or over-represent* or "over represent" or "over represented") adj3 (community or cultural* or demographic* or ethnic* or group or population))).mp.

54 ((care or case or client or discharge) adj3 (co-ordinat* or coordinat* or management or plan*)).mp.

55 (community adj3 (accommodat* or access* or agenc* or care or coach* or corrections or counsel* or discharge or education* or engagement or follow-up or goal* or hous* or integrat* or intervention or life skill* or linkage* or maintenance or manag* or measure* or mental health or monitor* or plan* or placement* or prevent* or program* or recover* or rehab* or reintegration or re-integration or reentry or re-entry or resettlement or re-settlement or resource* or therapy or therapies or service* or supervis* or support* or therap* or triag* or transition* or treatment or workshop*)).mp.

56 (aftercare or after-care).mp.

57 ((continu* or on-going or ongoing or through or transition*) adj1 care).mp.

58 (reintegration or re-integration or reentry or re-entry or resettlement or re-settlement).mp.

59 (counsel* or intervention* or therapy or therapies or treatment).mp. /freq=3 or "conditions of psychiatric treatment".mp.

60 ("Critical time intervention" or ((RESET adj (intervention or program*)) or pre-release plan* or "Connections Program" or (ENGAGER adj3 Intervention))).mp.

61 ("half-way house" or residential facilit*).mp.

62 ("in-reach" or inreach or "in reach").mp.

63 ((mental* or psychiatr* or psychological) adj3 (service* or center* or centre*)).mp.

64 (one stop shop* or one-stop shop*).mp.

65 (peer adj1 (group or groups or navigation or support)).mp.

66 (pre-discharge or predischarge or "pre discharge").mp.

67 (pre-release or prerelease or "pre-release").mp.

68 (post-discharge or postdischarge or "post discharge").mp.

69 (wraparound or wrap-around).mp.

70 or/1-18

71 or/19-38

72 39 and (or/40-49)

73 (acquit or acquitted or acquittal or bail or "leaving jail" or (discharg* adj3 (correction* or custody or detention or incarcerat* or jail or prison*)) or parole* or probation or ((re-enter* or re-entre* or reenter* or reentre*) adj5 (community or communities or public or society)) or ((release or released) adj3 (correction* or custody or detention or incarcerat* or jail or prison*)) or ((reintegrat* or re-integrat* or re-settle* or resettle*) adj3 (community or communities or public or society)) or (transition* adj5 (correction* or custody or detention or incarcerat* or jail or prison* or community or communities or public or society))).mp. adj8 (or/50-69)

74 70 and 71 and (72 or 73)

**APA PsycInfo <1806 to May Week 5 2024>**

1 Mental Health/

2 exp Mentally Ill Offenders/

3 exp Psychiatry/

4 exp Forensic Psychiatry/

5 forensic*.mp.

6 ((mental* or psychiatr* or psychological*) adj3 (condition* or diagnos?s* or disorder* or health* or hygiene or ill* or safe* or wellbeing or well-being or wellness)).mp.

7 ((mental* or psychiatr* or psychological*) adj3 (complex or comorbid* or highly distress* or high needs* or impair* or intense or persisten* or re-admit* or readmit* or recurrence or recurrent or reoccur* or re-occur* or serious* or sever* or treatment resistant* or treatment-resistant)).mp.

8 exp schizophrenia/

9 schizo*.mp.

10 dementia praecox.mp.

11 exp major depression/

12 (major adj depressi* adj disorder*).mp.

13 (involutional adj (depressi* or melancholia or paraphrenia* or psychos?s*)).mp.

14 exp Bipolar Disorder/

15 bipolar*.mp.

16 ((manic adj (disorder* or depress*)) or mania).mp.

17 psychos?s*.mp.

18 criminals/

19 criminal*.mp.

20 exp Prisoners/

21 Prisons/

22 prison*.mp.

23 penitentiar*.mp.

24 gaol*.mp.

25 jail*.mp.

26 (convict or convicts or convicted).mp.

27 offender*.mp.

28 remand*.mp.

29 sentence*.mp.

30 incarcerat*.mp.

31 (inmate or inmates or imprison*).mp.

32 (detain* not (immigrants or migrant* or refugee*)).mp.

33 ((detention adj3 (centre or center or facility or facilities or institution*)) not (immigrants or migrant* or refugee*)).mp.

34 forensic*.mp.

35 (pre-trial or pretrial or "pre trial").mp.

36 ("under trial" or under-trial or undertrial).mp.

37 (correction* adj3 (centre or center or facility or facilities or institution*)).mp. not (author correction or "correction to" or "Corrections To").ti.

38 (acquit or acquitted or acquittal or bail or "leaving jail" or (discharg* adj3 (correction* or custody or detention or incarcerat* or jail or prison*)) or parole* or probation or ((re-enter* or re-entre* or reenter* or reentre*) adj5 (community or communities or public or society)) or ((release or released) adj3 (correction* or custody or detention or incarcerat* or jail or prison*)) or ((reintegrat* or re-integrat* or re-settle* or resettle*) adj3 (community or communities or public or society)) or (transition* adj5 (correction* or custody or detention or incarcerat* or jail or prison* or community or communities or public or society))).mp.

39 Case Management/

40 Community Mental Health Centers/

41 Community Mental Health Services/

42 Mental Health Services/

43 peers/

44 exp psychosocial rehabilitation/

45 Rehabilitation/

46 exp Social Support/

47 Psychiatric Social Workers/

48 exp Therapeutic Processes/

49 ((alternative* or mental* or psychosocial* or psycho-social* or psychoeducation* or psycho-education* or resilient or resilience* or self efficacy or social or strength* based or wellbeing or well being or vocational or bridge or bridging or dual diagnos?s* or reentry) adj3 (care or counsel* or intervention* or maintenance or model or models or plan* or practice* or program* or service* or support* or system therapy or therapies or training or treatment or workshop*)).mp.

50 assertive community treatment.mp.

51 ((community or social or public) adj3 integrat*).mp.

52 (Aboriginal* or "First Nation" or "First Nations" or Indian or Indigenous or Inuit or Metis or Native or Natives or (Afro* or Afric* or Black or Blacks or ethnocultural or racialized) or (cultural* adj3 (appropriate* or approach or relevant* or specific*)) or ((overrepresent* or over-represent* or "over represent" or "over represented") adj3 (community or cultural* or demographic* or ethnic* or group or population))).mp.

53 ((care or case or client or discharge) adj3 (co-ordinat* or coordinat* or management or plan*)).mp.

54 (community adj3 (accommodat* or access* or agenc* or care or coach* or corrections or counsel* or discharge or education* or engagement or follow-up or goal* or hous* or integrat* or intervention or life skill* or linkage* or maintenance or manag* or measure* or mental health or monitor* or plan* or placement* or prevent* or program* or recover* or rehab* or reintegration or re-integration or reentry or re-entry or resettlement or re-settlement or resource* or therapy or therapies or service* or supervis* or support* or therap* or triag* or transition* or treatment or workshop*)).mp.

55 (aftercare or after-care).mp.

56 ((continu* or on-going or ongoing or through or transition*) adj1 care).mp.

57 (reintegration or re-integration or reentry or re-entry or resettlement or re-settlement).mp.

58 (counsel* or intervention* or therapy or therapies or treatment).mp. /freq=3 or "conditions of psychiatric treatment".mp.

59 ("Critical time intervention" or ((RESET adj (intervention or program*)) or pre-release plan* or "Connections Program" or (ENGAGER adj3 Intervention))).mp.

60 ("half-way house" or residential facilit*).mp.

61 ("in-reach" or inreach or "in reach").mp.

62 ((mental* or psychiatr* or psychological) adj3 (service* or center* or centre*)).mp.

63 (one stop shop* or one-stop shop*).mp.

64 (peer adj1 (group or groups or navigation or support)).mp.

65 (pre-discharge or predischarge or "pre discharge").mp.

66 (pre-release or prerelease or "pre-release").mp.

67 (post-discharge or postdischarge or "post discharge").mp.

68 (wraparound or wrap-around).mp.

69 or/1-17

70 or/18-37

71 38 and (or/39-48)

72 (acquit or acquitted or acquittal or bail or "leaving jail" or (discharg* adj3 (correction* or custody or detention or incarcerat* or jail or prison*)) or parole* or probation or ((re-enter* or re-entre* or reenter* or reentre*) adj5 (community or communities or public or society)) or ((release or released) adj3 (correction* or custody or detention or incarcerat* or jail or prison*)) or ((reintegrat* or re-integrat* or re-settle* or resettle*) adj3 (community or communities or public or society)) or (transition* adj5 (correction* or custody or detention or incarcerat* or jail or prison* or community or communities or public or society))).mp. adj8 (or/49-68)

73 69 and 70 and (71 or 72)

**Embase Classic+Embase <1947 to 2024 June 05>**

1 mental health/

2 exp Mental Disorders/

3 Mentally Ill Persons/

4 exp Psychiatry/

5 exp Forensic Psychiatry/

6 forensic*.mp.

7 ((mental* or psychiatr* or psychological*) adj3 (condition* or diagnos?s* or disorder* or health* or hygiene or ill* or safe* or wellbeing or well-being or wellness)).mp.

8 ((mental* or psychiatr* or psychological*) adj3 (complex or comorbid* or highly distress* or high needs* or impair* or intense or persisten* or re-admit* or readmit* or recurrence or recurrent or reoccur* or re-occur* or serious* or sever* or treatment resistant* or treatment-resistant)).mp.

9 schizophrenia/ or schizophrenia spectrum disorder/

10 exp Schizophrenia/

11 schizo*.mp.

12 Depressive Disorder, Major/

13 (major adj depressi* adj disorder*).mp.

14 (involutional adj (depressi* or melancholia or paraphernalia* or psychos?s)).mp.

15 exp Bipolar Disorder/

16 bipolar*.mp.

17 (manic adj (disorder* or depress*)).mp.

18 psychos?s*.mp.

19 criminals/

20 criminal*.mp.

21 exp Prisoners/

22 Prisons/

23 prison*.mp.

24 penitentiar*.mp.

25 gaol*.mp.

26 jail*.mp.

27 (convict or convicts or convicted).mp.

28 offender*.mp.

29 remand*.mp.

30 sentence*.mp.

31 incarcerat*.mp.

32 (inmate or inmates or imprison*).mp.

33 (detain* not (immigrants or migrant* or refugee*)).mp.

34 ((detention adj3 (centre or center or facility or facilities or institution*)) not (immigrants or migrant* or refugee*)).mp.

35 forensic*.mp.

36 (pre-trial or pretrial or "pre trial").mp.

37 ("under trial" or under-trial or undertrial).mp.

38 (correction* adj3 (centre or center or facility or facilities or institution*)).mp. not (author correction or "correction to" or "Corrections To").ti.

39 (acquit or acquitted or acquittal or bail or "leaving jail" or (discharg* adj3 (correction* or custody or detention or incarcerat* or jail or prison*)) or parole* or probation or ((re-enter* or re-entre* or reenter* or reentre*) adj5 (community or communities or public or society)) or ((release or released) adj3 (correction* or custody or detention or incarcerat* or jail or prison*)) or ((reintegrat* or re-integrat* or re-settle* or resettle*) adj3 (community or communities or public or society)) or (transition* adj5 (correction* or custody or detention or incarcerat* or jail or prison* or community or communities or public or society))).mp.

40 Case Management/

41 Community Mental Health Centers/

42 Community Mental Health Services/

43 Mental Health Services/

44 Peer Group/

45 Psychiatric Rehabilitation/

46 Rehabilitation/

47 exp Social Support/

48 Social Work, Psychiatric/

49 Therapeutics/

50 ((alternative* or mental* or psychosocial* or psycho-social* or psychoeducation* or psycho-education* or resilient or resilience* or self efficacy or social or strength* based or wellbeing or well being or vocational or bridge or bridging or dual diagnos?s* or reentry) adj3 (care or counsel* or intervention* or maintenance or model or models or plan* or practice* or program* or service* or support* or system therapy or therapies or training or treatment or workshop*)).mp.

51 assertive community treatment.mp.

52 ((community or social or public) adj3 integrat*).mp.

53 (Aboriginal* or "First Nation" or "First Nations" or Indian or Indigenous or Inuit or Metis or Native or Natives or (Afro* or Afric* or Black or Blacks or ethnocultural or racialized) or (cultural* adj3 (appropriate* or approach or relevant* or specific*)) or ((overrepresent* or over-represent* or "over represent" or "over represented") adj3 (community or cultural* or demographic* or ethnic* or group or population))).mp.

54 ((care or case or client or discharge) adj3 (co-ordinat* or coordinat* or management or plan*)).mp.

55 (community adj3 (accommodat* or access* or agenc* or care or coach* or corrections or counsel* or discharge or education* or engagement or follow-up or goal* or hous* or integrat* or intervention or life skill* or linkage* or maintenance or manag* or measure* or mental health or monitor* or plan* or placement* or prevent* or program* or recover* or rehab* or reintegration or re-integration or reentry or re-entry or resettlement or re-settlement or resource* or therapy or therapies or service* or supervis* or support* or therap* or triag* or transition* or treatment or workshop*)).mp.

56 (aftercare or after-care).mp.

57 ((continu* or on-going or ongoing or through or transition*) adj1 care).mp.

58 (reintegration or re-integration or reentry or re-entry or resettlement or re-settlement).mp.

59 (counsel* or intervention* or therapy or therapies or treatment).mp. /freq=3 or "conditions of psychiatric treatment".mp.

60 ("Critical time intervention" or ((RESET adj (intervention or program*)) or pre-release plan* or "Connections Program" or (ENGAGER adj3 Intervention))).mp.

61 ("half-way house" or residential facilit*).mp.

62 ("in-reach" or inreach or "in reach").mp.

63 ((mental* or psychiatr* or psychological) adj3 (service* or center* or centre*)).mp.

64 (one stop shop* or one-stop shop*).mp.

65 (peer adj1 (group or groups or navigation or support)).mp.

66 (pre-discharge or predischarge or "pre discharge").mp.

67 (pre-release or prerelease or "pre-release").mp.

68 (post-discharge or postdischarge or "post discharge").mp.

69 (wraparound or wrap-around).mp.

70 or/1-18

71 or/19-38

72 39 and (or/40-49) [release terms AND reintegration subject headings]

73 (acquit or acquitted or acquittal or bail or "leaving jail" or (discharg* adj3 (correction* or custody or detention or incarcerat* or jail or prison*)) or parole* or probation or ((re-enter* or re-entre* or reenter* or reentre*) adj5 (community or communities or public or society)) or ((release or released) adj3 (correction* or custody or detention or incarcerat* or jail or prison*)) or ((reintegrat* or re-integrat* or re-settle* or resettle*) adj3 (community or communities or public or society)) or (transition* adj5 (correction* or custody or detention or incarcerat* or jail or prison* or community or communities or public or society))).mp. adj8 (or/50-69) [release terms ADJ8 reintegration programs/terms]

74 70 and 71 and (72 or 73)

**EBSCO CINAHL**

# Query

S69 S66 AND S67 AND S68

S68 ( ( S37 OR S38 OR S39 OR S40 OR S41 OR S42 OR S43 OR S44 OR S45 ) AND 65 ) OR ( (S46 OR S47 OR S48 OR S49 OR S50 OR S51 OR S52 OR S53 OR S54 OR S55 OR S56 OR S57 OR S58 OR S59 OR S60 OR S61 OR S62 OR 63) N8 S65 )

S67 S18 OR S19 OR S20 OR S21 OR S22 OR S23 OR S24 OR S25 OR S26 OR S27 OR S28 OR S29 OR S30 OR S31 OR S32 OR S33 OR S34 OR S35 OR S36

S66 S1 OR S2 OR S3 OR S4 OR S5 OR S6 OR S7 OR S8 OR S9 OR S10 OR S11 OR S12 OR S13 OR S14 OR S15 OR S16 OR S17

S65 (acquit or acquitted or acquittal or bail or "leaving jail" or (discharg* N3 (correction* or custody or detention or incarcerat* or jail or prison*)) or parole* or probation or ((re-enter* or re-entre* or reenter* or reentre*) N5 (community or communities or public or society)) or ((release or released) N3 (correction* or custody or detention or incarcerat* or jail or prison*)) or ((reintegrat* or re-integrat* or resettle* or resettle*) N3 (community or communities or public or society)) or (transition* N5 (correction* or custody or detention or incarcerat* or jail or prison* or community or communities or public or society)))

S64 (wraparound or wrap-around)

S63 (post-discharge or postdischarge or "post discharge")

S62 (pre-release or prerelease or "pre-release")

S61 (pre-discharge or predischarge or "pre discharge")

S60 (peer group or peer groups)

S59 (one stop shop* or one-stop shop*)

S58 ((mental* or psycholog* or psychosocial or psychiatr*) N3 (care or cares rehabilitat*))

S57 ("mental health services" or "mental health service")

S56 (mental N3 (care or intervention* or model or models or program* or therapy or therapies or treatment* or service* or system*)

S55 ("in-reach" or inreach or "in reach")

S54 ("half-way house" or residential facilit*)

S53 ("Critical time intervention" or ((RESET N1 (intervention or program*)) or pre-release plan* or "Connections Program" or (ENGAGER N3 Intervention))

S52 TI ( (counsel* or intervention* or therapy or therapies or treatment) ) OR AB ( (counsel* or intervention* or therapy or therapies or treatment) )

S51 (aftercare or after-care) OR (reintegration or re-integration or reentry or re-entry or resettlement or re-settlement) or ((continu* or on-going or ongoing or through or transition*) N1 care)

S50 (community N3 (accommodat* or access* or aftercare or after care or agenc* or care or coach* or corrections or counsel* or discharge or education* or engagement or follow-up or goal* or hous* or integrat* or intervention or life skill* or linkage* or maintenance or manag* or measure* or mental health or monitor* or plan* or placement* or prevent* or program* or recover* or rehab* or reintegration or re-integration or reentry or re-entry or resettlement or re-settlement or resource* or therapy or therapies or service* or supervis* or support* or therap* or triag* or transition* or treatment or workshop*))

S49 (community mental health centre* or community mental health center)

S48 (assertive community treatment) OR ((care or case or client or discharge) N3 (co-ordinat* or coordinat* or management or plan*)) OR ((community or social or public) N3 integrat*)

S47 (Aboriginal* or "First Nation" or "First Nations" or Indian or Indigenous or Inuit or Metis or Native or Natives or (Afro* or Afric* or Black or Blacks or ethnocultural or racialized) or (cultural* N3 (appropriate* or approach or relevant* or specific*)) or ((overrepresent* or over-represent* or "over represent" or "over represented") N3 (community or cultural* or demographic* or ethnic* or group or population)))

S46 ((alternative* or mental* or psychosocial* or psycho-social* or psychoeducation* or psychoeducation* or resilient or resilience* or self efficacy or social or strength* based or wellbeing or well being or vocational or bridge or bridging or dual diagnos?s* or reentry) N3 (care or counsel* or intervention* or maintenance or model or models or plan* or practice* or program* or service* or support* or system therapy or therapies or training or treatment or workshop*))

S45 (MH "Therapeutics")

S44 (MH "Social Work, Psychiatric")

S43 (MH "Support, Social+")

S42 (MH "Rehabilitation Centers")

S41 (MH "Rehabilitation, Psychosocial")

S40 (MH "Peer Group")

S39 (MH "Mental Health Services")

S38 (MH "Community Mental Health Services")

S37 (MH "Case Management")

S36 ( (correction* N3 (centre or center or facility or facilities or institution*)) ) NOT ( (author correction or "correction to" or "Corrections To") )

S35 ("under trial" or under-trial or undertrial)

S34 (pre-trial or pretrial or "pre trial")

S33 forensic*

S32 ((detention N3 (centre or center or facility or facilities or institution*)) not (immigrants or migrant* or refugee*))

S31 (detain* not (immigrants or migrant* or refugee*))

S30 (inmate or inmates or imprison*)

S29 incarcerat*

S28 sentence

S27 remand*

S26 offender*

S25 (convict or convicts or convicted)

S24 jail*

S23 gaol*

S22 penitentiar*

S21 prison*

S20 (MM "Prisoners")

S19 "criminal*"

S18 (MM "Public Offenders")

S17 psychos?s*

S16 (manic N1 (disorder* or depress*))

S15 bipolar*

S14 (MH "Bipolar Disorder")

S13 (involutional N1 (depressi* or melancholia or paraphrenia* or psychos?s))

S12 (major N1 depressi* N1 disorder*)

S11 "dementia praecox"

S10 schizo*

S9 (MH "Schizophrenia+")

S8 ((mental* or psychiatr* or psychological*) N3 (complex or comorbid* or highly distress* or high needs* or impair* or intense or persisten* or re-admit* or readmit* or recurrence or recurrent or reoccur* or re-occur* or serious* or sever* or treatment resistant* or treatment-resistant))

S7 ((mental* or psychiatr* or psychological*) N3 (condition* or diagnos?s* or disorder* or health* or hygiene or ill* or safe* or wellbeing or well-being or wellness))

S6 "forensic*"

S5 (MM "Forensic Psychiatry")

S4 (MH "Psychiatry")

S3 (MM "Mentally Ill Offenders")

S2 (MM "Mental Disorders")

S1 MM "Mental Health"

**Ebsco Criminal Justice Abstracts**

| S50 | S47 AND S48 AND S49 |
| --- | --- |
| S49 | (S27 OR S28 OR S29 OR S30 ORS31 OR S32 OR S33 OR S34 ORS35 OR S36 OR S37 OR S38 ORS39 OR S40 OR S41 OR S42 ORS43 OR S44 OR S45) AND ((acquit or acquitted or acquittal or bail or "leaving jail" or (discharg* N3(correction* or custody or detention or incarcerat* or jailor prison*)) or parole* or probation or ((re-enter*or re-entre* or reenter* or reentre*) N10 (community or communities or public or society)) or ((release or released) N3 (correction* or custody or detention or incarcerat*or jail or prison*)) or ((reintegrat* or re-integrat* or re-settle* or resettle*)N3 (community or communities or public or society)) or (transition* N5(correction* or custody or detention or incarcerat* or jail or prison* or community or communities or public or society))) |
| S48 | S11 OR S12 OR S13 OR S14 ORS15 OR S16 OR S17 OR S18 ORS19 OR S20 OR S21 OR S22 ORS23 OR S24 OR S25 OR S26 |
| S47 | S1 OR S2 OR S3 OR S4 OR S5OR S6 OR S7 OR S8 OR S9 ORS10 |
| S46 | (S27 OR S28 OR S29 OR S30 ORS31 OR S32 OR S33 OR S34 ORS35 OR S36 OR S37 OR S38 ORS39 OR S40 OR S41 OR S42 ORS43 OR S44 OR S45) N10 ((acquit or acquitted or acquittal or bail or "leaving jail" or (discharg* N3(correction* or custody or detention or incarcerat* or jailor prison*)) or parole* or probation or ((re-enter*or re-entre* or reenter* or reentre*)N10 (community or communities or public or society)) or ((release or released) N3 (correction* or custody or detention or incarcerat*or jail or prison*)) or ((reintegrat* or re-integrat* or re-settle* or resettle*)N3 (community or communities or public or society)) or (transition* N5 (correction* or custody or detention or incarcerat* or jail or prison* or community or communities or public or society))) |
| S45 | (wraparound or wrap-around) |
| S44 | (post-discharge or postdischarge or "post discharge") |
| S43 | (pre-release or prerelease or "pre-release") |
| S42 | (pre-discharge or predischarge or "pre discharge") |
| S41 | (peer group or peer groups) |
| S40 | (one stop shop* or one-stop shop*) |
| S39 | ((mental* or psycholog* or psychosocial or psychiatr*) N3 (care or cares rehabilitat*)) |
| S38 | ("mental health services" or "mental health service") |
| S37 | (mental N3 (care or intervention* or model or models or program* or therapy or therapies or treatment*or service* or system*)) |
| S36 | ("in-reach" or inreach or "in reach") |
| S35 | ("half-way house" or residential facilit*) |
| S34 | ("Critical time intervention" or((RESET N1 (intervention or program*)) or pre-release plan* or "Connections Program" or(ENGAGER N3 Intervention))) |
| S33 | TI ( (counsel* or intervention* or therapy or therapies or treatment) )OR AB ( (counsel* or intervention*or therapy or therapies or treatment) ) |
| S32 | ((continu* or on-going or ongoing or through or transition*) N1 care) |
| S31 | (community N3 (accommodat* or access* or aftercare or after care or agenc* or care or coach* or corrections or counsel* or discharge or education* or engagement or follow-up or goal* or hous* or integrat* or intervention or life skill* or linkage* or maintenance or manag* or measure* or mental health or monitor* or plan* or placement* or prevent* or program* or recover* or rehab* or reintegration or re-integration or reentry or re-entry or resettlement or re-settlement or resource* or therapy or therapies or service* or supervis* or support* or therap* or triag* or transition* or treatment or workshop*)) |
| S30 | (community mental health centre*or community mental health center) |
| S29 | (assertive community treatment)OR((care or case or client or discharge) N3 (co-ordinat* or coordinat* or management or plan*)) OR ((community or social or public) N3 integrat*) |
| S28 | (Aboriginal* or "First Nation" or "First Nations" or Indian or Indigenous or Inuit or Metis or Native or Natives or (Afro* or Afric*or Black or Blacks or ethnocultural or racialized) or (cultural*N3(appropriate* or approach or relevant* or specific*)) or((overrepresent* or over-represent*or "over represent" or "overrepresented") N3 (community or cultural* or demographic* or ethnic*or group or population))) |
| S27 | ((alternative* or mental* or psychosocial* or psycho-social* or psychoeducation* or psycho-education* or resilient or resilience*or self efficacy or social or strength* based or wellbeing or well being or vocational or bridge or bridging or dual diagnos?s* or reentry) N3 (care or counsel* or intervention* or maintenance or model or models or plan* or practice* or program* or service* or support* system or therapy or therapies or training or treatment or workshop*)) |
| S26 | ( (correction* N3 (centre or center or facility or facilities or institution*))) NOT ( (author correction or "correction to" or "Corrections To") ) |
| S25 | ("under trial" or under-trial or undertrial) |
| S24 | (pre-trial or pretrial or "pre trial") |
| S23 | forensic* |
| S22 | ((detention N3 (centre or center or facility or facilities or institution*))not (immigrants or migrant* or refugee*)) |
| S21 | (detain* not (immigrants or migrant*or refugee*)) |
| S20 | (inmate or inmates or imprison*) |
| S19 | incarcerat* |
| S18 | sentence* |
| S17 | offender* OR remand* |
| S16 | (convict or convicts or convicted) |
| S15 | jail* |
| S14 | gaol* |
| S13 | penitentiar* |
| S12 | prison* |
| S11 | criminal* |
| S10 | psychos?s* |
| S9 | (manic N1 (disorder* or depress*)) |
| S8 | bipolar* |
| S7 | (involutional N1 (depressi* or melancholia or paraphrenia* or psychos?s)) |
| S6 | (major N1 depressi* N1 disorder*) |
| S50 | psychos?s* |
| S4 | schizo* |
| S3 | ((mental* or psychiatr* or psychological*) N3 (complex or comorbid* or highly distress* or high needs* or impair* or intense or persisten* or re-admit* or readmit*or recurrence or recurrent or reoccur* or re-occur* or serious* or sever* or treatment resistant* or treatment-resistant)) |
| S2 | ((mental* or psychiatr* or psychological*) N3 (condition* or diagnos?s* or disorder* or health*or hygiene or ill* or safe* or wellbeing or well-being or wellness)) |
| S1 | forensic* |

**ProQuest Applied Social Sciences Index & Abstracts (ASSIA)**

abstract(forensic OR mental* OR psychiatr* OR psychological* OR schizo* OR "Major depression" OR bipolar* OR psychos?s*) AND abstract(criminal* OR prison* OR penitentiar* OR gaol* OR jail* OR convict OR convicts OR convicted offender* OR remand* OR sentence* OR incarcerat* OR inmate OR inmates OR imprison* OR detain* OR detention* OR forensic* OR pre-trial OR pretrial OR "pre trial" OR "under trial" OR under-trial OR undertrial OR correction* OR (Aboriginal* OR "First Nation" OR "First Nations" OR Indian OR Indigenous OR Inuit OR Metis OR Native OR Natives OR (Afro* OR Afric* OR Black OR Blacks OR ethnocultural OR racialized) OR (cultural* NEAR/3 (appropriate* OR approach OR relevant* OR specific*)) OR ((overrepresent* OR over-represent* OR "over represent" OR "over represented") NEAR/3 (community OR cultural* OR demographic* OR ethnic* OR group OR population)))) AND abstract(alternative* OR psychosocial* OR psycho-social* OR psychoeducation* OR psycho-education* OR re-integration OR reintegration OR resilient OR self efficacy OR social OR strength* based OR wellbeing OR well being OR vocational OR bridge OR bridging OR reentry OR "assertive community treatment" OR "community mental health" OR "Critical time intervention" OR RESET OR pre-release plan* OR "Connections Program" OR ENGAGER OR "half-way house" OR residential facilit* OR "in-reach" OR inreach OR "in reach" OR one stop shop* OR one-stop shop* OR "peer group" OR "peer groups" OR pre-discharge OR predischarge OR "pre discharge" OR pre-release OR prerelease OR "pre-release" OR post-discharge OR postdischarge OR wraparound OR wrap-around) AND abstract(acquit OR acquitted OR acquittal OR bail OR "leaving jail" OR parole* OR probation OR e-enter* OR re-entre* OR reenter* OR reentre* OR release OR released OR reintegrat* OR re-integrat* OR re-settle* OR resettle*) NOT (("author correction" OR "correction to" OR "Corrections To") OR (immigrants OR migrant* OR refugee*)) Applied Social Sciences Index & Abstracts (ASSIA)

**EBM Reviews - Cochrane Central Register of Controlled Trials <May 2024>**

1 Mental Health/

2 exp Mental Disorders/

3 Mentally Ill Persons/

4 exp Psychiatry/

5 exp Forensic Psychiatry/

6 forensic*.mp.

7 ((mental* or psychiatr* or psychological*) adj3 (condition* or diagnos?s* or disorder* or health* or hygiene or ill* or safe* or wellbeing or well-being or wellness)).mp.

8 ((mental* or psychiatr* or psychological*) adj3 (complex or comorbid* or highly distress* or high needs* or impair* or intense or persisten* or re-admit* or readmit* or recurrence or recurrent or reoccur* or re-occur* or serious* or sever* or treatment resistant* or treatment-resistant)).mp.

9 exp "Schizophrenia Spectrum and Other Psychotic Disorders"/

10 exp Schizophrenia/

11 schizo*.mp.

12 dementia praecox.mp.

13 Depressive Disorder, Major/

14 (major adj depressi* adj disorder*).mp.

15 (involutional adj (depressi* or melancholia or paraphrenia* or psychos?s)).mp.

16 exp Bipolar Disorder/

17 bipolar*.mp.

18 (manic adj (disorder* or depress*)).mp.

19 psychos?s*.mp.

20 criminals/

21 criminal*.mp.

22 exp Prisoners/

23 Prisons/

24 prison*.mp.

25 penitentiar*.mp.

26 gaol*.mp.

27 jail*.mp.

28 (convict or convicts or convicted).mp.

29 offender*.mp.

30 remand*.mp.

31 sentence*.mp.

32 incarcerat*.mp.

33 (inmate or inmates).mp.

34 (detain* not (immigrants or migrant* or refugee*)).mp.

35 ((detention adj3 (centre or center or facility or facilities or institution*)) not (immigrants or migrant* or refugee*)).mp.

36 forensic*.mp.

37 (pre-trial or pretrial or "pre trial").mp.

38 ("under trial" or under-trial or undertrial).mp.

39 (correction* adj3 (centre or center or facility or facilities or institution*)).mp. not (author correction or "correction to" or "Corrections To").ti.

40 Case Management/

41 Community Mental Health Centers/

42 Community Mental Health Services/

43 Mental Health Services/

44 Peer Group/

45 Psychiatric Rehabilitation/

46 Rehabilitation/

47 exp Social Support/

48 Social Work, Psychiatric/

49 Therapeutics/

50 ((alternative* or mental* or psychosocial* or psycho-social* or psychoeducation* or psycho-education* or resilient or resilience* or self efficacy or social or strength* based or wellbeing or well being or vocational or bridge or bridging or dual diagnos?s* or reentry) adj3 (care or counsel* or intervention* or maintenance or model or models or plan* or practice* or program* or service* or support* or system therapy or therapies or training or treatment or workshop*)).mp.

51 assertive community treatment.mp.

52 ((community or social or public) adj3 integrat*).mp.

53 (Aboriginal* or "First Nation" or "First Nations" or Indian or Indigenous or Inuit or Metis or Native or Natives or (Afro* or Afric* or Black or Blacks or ethnocultural or racialized) or (cultural* adj3 (appropriate* or approach or relevant* or specific*)) or ((overrepresent* or over-represent* or "over represent" or "over represented") adj3 (community or cultural* or demographic* or ethnic* or group or population))).mp.

54 ((care or case or client or discharge) adj3 (co-ordinat* or coordinat* or management or plan*)).mp.

55 (community mental health centre* or community mental health center).mp.

56 (community adj3 (accommodat* or access* or aftercare or after care or agenc* or care or coach* or corrections or counsel* or discharge or education* or engagement or follow-up or goal* or hous* or integrat* or intervention or life skill* or linkage* or maintenance or manag* or measure* or mental health or monitor* or plan* or placement* or prevent* or program* or recover* or rehab* or reintegration or re-integration or reentry or re-entry or resettlement or re-settlement or resource* or therapy or therapies or service* or supervis* or support* or therap* or triag* or transition* or treatment or workshop*)).mp.

57 (aftercare or "after care").mp.

58 ((continu* or on-going or ongoing or through or transition*) adj1 care).mp.

59 (reintegration or re-integration or reentry or re-entry or resettlement or re-settlement).mp.

60 (counsel* or intervention* or therapy or therapies or treatment).mp. /freq=3 or "conditions of psychiatric treatment".mp.

61 ("Critical time intervention" or ((RESET adj (intervention or program*)) or pre-release plan* or "Connections Program" or (ENGAGER adj3 Intervention))).mp.

62 ("half-way house" or residential facilit*).mp.

63 ("in-reach" or inreach or "in reach").mp.

64 ((mental* or psychiatr* or psychological) adj3 (service* or center* or centre*)).mp.

65 (one stop shop* or one-stop shop*).mp.

66 (peer adj1 (group or groups or navigation or support)).mp.

67 (pre-discharge or predischarge or "pre discharge").mp.

68 (pre-release or prerelease or "pre-release").mp.

69 (post-discharge or postdischarge or "post discharge").mp.

70 (wraparound or wrap-around).mp.

71 (acquit or acquitted or acquittal or bail or "leaving jail" or (discharg* adj3 (correction* or custody or detention or incarcerat* or jail or prison*)) or parole* or probation or ((re-enter* or re-entre* or reenter* or reentre*) adj5 (community or communities or public or society)) or ((release or released) adj3 (correction* or custody or detention or incarcerat* or jail or prison*)) or ((reintegrat* or re-integrat* or re-settle* or resettle*) adj3 (community or communities or public or society)) or (transition* adj5 (correction* or custody or detention or incarcerat* or jail or prison* or community or communities or public or society))).mp. adj8 (or/50-69) [release terms ADJ8 reintegration programs/terms]

72 or/1-19 kjj

73 or/20-39

74 (or/40-49) and 71

75 (or/50-70) and 71

76 72 and 73 and (74 or 75)
