# Supplementary material for: Reintegration programmes for people with severe mental illnesses released from correctional institutions: systematic review
Source: BJPsych Open. 2026 Jul 8;12(4):e181. doi: 10.1192/bjo.2026.12034 (PMC13359055; doi:10.1192/bjo.2026.12034)
Supplement: Simpson et al. supplementary material 2 — Simpson et al. supplementary material [file S2056472426120341sup002.docx]

**Appendix B**

Definitions of serious mental illness (SMI) and recidivism.

| **Study** | **Serious Mental Illness (SMI)** | **Recidivism** |
| --- | --- | --- |
| Bellamy et al. 2019 (18) | 9% schizophrenia, 53% bipolar disorder, and 48% major depression. 46% also diagnosed with anxiety. | Defined as reincarceration (any return to jail). |
| Carr et al. 2016 (19) | 60% primary diagnosis of a psychotic disorder, such as schizophrenia or schizoaffective disorder; 18% diagnosed with bipolar disorder. 78% diagnosed with a substance use disorder, mostly polysubstance dependence (24%). | Defined in terms of reconviction rates. |
| Shaw et al. 2017 (27) | Schizophrenia was the commonest diagnosis in both the CTI (71%) and TAU (73%) groups; 13% overall had major depressive disorder (MDD) and 8% schizoaffective/schizophreniform disorder. | Planned to measure this by tracking criminal justice contact and reconviction at 12 months post-release from prison, but collection and analysis postponed and not reported. |
| Smith et al. 2018 (33) | Two thirds had diagnoses of schizophrenia, schizotypal and delusional disorders (58.1%) or bipolar affective disorder (2.3%). Additional 16.3% had a drug induced psychotic episode. | Defined as reincarceration. |
| Comartin et al. 2022 (20) | Mentions that all participants had a mental illness, a requirement for participation in the Mental Health Jail Interventions (MHJIs). 79.4% had co-occurring mental illness and substance use disorder (SUD). | Return to prison due to technical violation or new offence. |
| Green et al. 2016 (37) | Psychotic disorder: TR-long (69.6%), TR-short (56.7%), and TCP-only (80%). Other diagnoses included: mood disorders, anxiety, SUD, and personality disorder (PD). | Defined as reincarceration, including for parole breaches. |
| Hartwell et al. 2009 (41) | Inmates eligible if their axis I major mental illness resulted in significant functional impairment lasting ≥1 year. 74% of included participants received prior community mental health services: 53% for thought disorders, 40% for mood disorders. 6% were diagnosed with a PD. | Defined as reincarceration. |
| Held et al. 2012 (38) | 19.3% had schizophrenic disorders and other psychoses, 38.2% had affective disorders including depression, bipolar disorder, and other mood disorders. 9.7% had other mental disorders. Majority (87%) diagnosed with SUD. | Defined as annual bookings, charges, felonies, and misdemeanors. |
| Johnson et al. 2015 (35) | 22 incarcerated women diagnosed with SUD and MDD. | Assessed as drug using days, drinking days, and days incarcerated 6 months during post-release follow-up. |
| Lovell et al. 2005 (40) | 30% schizophrenia, 30% other thought disorders, 18% bipolar disorder, 7% depression, 15% other (dementia, brain injury, developmental disability, and severe PD). | Defined as reconviction for any new offence (felonies and misdemeanors). |
| MacInnes et al. 2021 (36) | RESET group: mood disorder (45%), schizophrenia (29%), PD (19%), and other (6%). Comparison group: mood disorder (53%), schizophrenia (23%), PD (10%) and other 4 (13%). Main “other” diagnosis was drug and/or alcohol misuse. | Assessed reoffending at 2 weeks, 3 and 9 months. |
| McKenna et al. 2015 (39) | 54% schizophrenia, 11% depression, 10% bipolar disorder, 3% head injury, 2% anxiety disorder, and 6% other. No diagnosis made in 15%. | Post-release reoffending: new charges and convictions. |
| Sacks et al. 2004 (24) | 78% had an “axis I mental disorder”, 37% antisocial personality disorder; 90% had SUD. | Reincarceration and criminal activity (any criminal activity or alcohol- or drug use-related criminal activity); restricted to new crimes only. |
| Sacks et al. 2012 (25) | Co-occurring SUD with either bipolar mood disorders, MDD, depressive disorders not otherwise specified, dysthymia, paranoid/delusional disorders, schizophrenic disorders, schizophreniform disorder, schizoaffective disorder, psychotic disorder not otherwise specified, substance induced psychotic disorder, brief reactive psychosis, dissociative identity disorder, cluster A personality disorders (schizoid, schizotypal, paranoid), and posttraumatic stress disorder. | Reincarceration for new offences; parole or technical violations excluded. |
| Solomon et al. 1994 (31) | 85.9% schizophrenia, 11.1% major affective disorder, 3% other (including unspecified psychotic disorder and PD). | Reincarceration encompassing both new criminal charges and technical violations of probation or parole. |
| Solomon & Draine 1995 (32) | 84% schizophrenia, 10.6% major affective disorder; 54.3% substance abuse involvement. | Defined as reincarceration. |
| Stewart et al. 2017 (34) | At least one of the following: moderate to severe impairment due to major mental illness (includes schizophrenia, schizoaffective disorders, MDD, and bipolar disorders), moderate to severe functional impairment due to personality disorder (excepting antisocial personality disorder), moderate to severe impairment due to organic brain dysfunction or acquired brain injury and moderate to severe impairment due to intellectual disability. | Reincarceration (to provincial or federal prisons) due to new offences or parole violations. Criminal recidivism categorised as general, violent, and sexual recidivism. |
| Sullivan et al. 2007 (26) | Approximately 90% diagnosed with substance use or dependence disorder; 77% diagnosed with at least one mental disorder. About 60% met diagnostic criteria for a SMI—schizophrenia, bipolar disorder, or major depression—and 38% diagnosed with antisocial PD. | Reincarceration and criminal activity. New criminal offences included, whereas technical and parole violations were excluded. |
| Taylor 2006 (28) | 33.5% schizophrenia/psychotic disorders, 31.1% MDD, 30.6% bipolar disorders, and 30.6% SUD. | Number or arrests (bookings), number of convictions, and days spent in jail. |
| Theurer & Lovell 2008 (29) | 56% psychotic disorder, 20% depression, 20% bipolar, and 3% other. 89% with co-occurring chemical dependency/abuse and 52% PD. | New felony convictions and rearrests. |
| Ventura et al. 1998 (30) | 33% PD, 22% schizophrenia, and 17% affective disorder; remaining 28% mostly had adjustment disorders. 72% with PD had comorbid SUD, 69% with affective disorder had SUD, 35% with schizophrenia had SUD, and 56% SUD for those with other diagnoses. | Defined as rearrest within Lucas County (Toledo, Ohio); potentially undercounting those who relocated. |
| Cuddeback et al. 2016 (21) | Diagnoses included schizophrenia, bipolar disorder, depression, and SUD. | Outcomes are limited to administrative approval for Medicaid, actual enrollment status, and the utilization of mental health services. |
| Domino et al. 2017 (43) | Diagnosis of schizophrenia or bipolar disorder. | Reincarceration due to technical violations or new charges. |
| Gertner et al. 2019 (22) | Diagnosis of schizophrenia or bipolar disorder (with or without SUD). | Examines the effect of referral to an expedited Medicaid enrollment program on the utilization of SUD treatment in individuals with SMI post-release. |
| Morrissey et al. 2016 (23) | Diagnosis of schizophrenia or bipolar disorder. | Reincarceration due to new charges or technical violations. |
| Wenzlow et al. 2011 (42) | Diagnosed as having major depression, bipolar disorder, or a psychotic illness and being identified as requiring intensive treatment to adjust to incarceration. | Measures effectiveness of a discharge planning program in increasing Medicaid enrollment and use of mental health services. |
